# Diffusion–based virtual MR elastography for predicting recurrence of solitary hepatocellular carcinoma after hepatectomy

**DOI:** 10.1186/s40644-024-00759-8

**Published:** 2024-08-13

**Authors:** Jiejun Chen, Wei Sun, Wentao Wang, Caixia Fu, Robert Grimm, Mengsu Zeng, Shengxiang Rao

**Affiliations:** 1 Department of Radiology, Zhongshan Hospital, Fudan University, No. 180 Fenglin Road, Xuhui District, Shanghai, 200032 China; 2 Shanghai Institute of Medical Imaging, Shanghai, China; 3 Department of Cancer Center, Zhongshan Hospital, Fudan University, Shanghai, China; 4 MR Application development, Siemens Shenzhen Magnetic Resonance Ltd, Shenzhen, China; 5 MR Application Predevelopment, Siemens Healthineers AG, Erlangen, Germany

## Abstract

**Background:**

To explore the capability of diffusion-based virtual MR elastography (vMRE) in the preoperative prediction of recurrence in hepatocellular carcinoma (HCC) and to investigate the underlying relevant histopathological characteristics.

**Methods:**

Between August 2015 and December 2016, patients underwent preoperative MRI examination with a dedicated DWI sequence (b-values: 200,1500 s/mm^2^) were recruited. The ADC values and diffusion-based virtual shear modulus (*μ*_*diff*_) of HCCs were calculated and MR morphological features were also analyzed. The Cox proportional hazards model was used to identify the risk factors associated with tumor recurrence. A preoperative radiologic model and postoperative model including pathological features were built to predict tumor recurrence after hepatectomy.

**Results:**

A total of 87 patients with solitary surgically confirmed HCCs were included in this study. Thirty-five patients (40.2%) were found to have tumor recurrence after hepatectomy. The preoperative model included higher *μ*_*diff*_ and corona enhancement, while the postoperative model included higher *μ*_*diff*_, microvascular invasion, and histologic tumor grade. These factors were identified as significant prognostic factors for recurrence-free survival (RFS) (all *p* < 0.05). The HCC patients with *μ*_*diff*_ values > 2.325 kPa showed poorer 5-year RFS after hepatectomy than patients with *μ*_*diff*_ values ≤ 2.325 kPa (*p* < 0.001). Moreover, the higher *μ*_*diff*_ values was correlated with the expression of CK19 (3.95 ± 2.37 vs. 3.15 ± 1.77, *p* = 0.017) and high Ki-67 labeling index (4.22 ± 1.63 vs. 2.72 ± 2.12, *p* = 0.001).

**Conclusions:**

The *μ*_*diff*_ values related to the expression of CK19 and Ki-67 labeling index potentially predict RFS after hepatectomy in HCC patients.

**Supplementary Information:**

The online version contains supplementary material available at 10.1186/s40644-024-00759-8.

## Introduction

Hepatocellular carcinoma (HCC) is the most common primary malignant tumor of the liver which ranked as the third leading cause of cancer-related death worldwide [1]. Although great progress has been made in the early diagnosis and surgical techniques of HCC, the prognosis of HCC is still very poor, the tumor recurrence rate is up to 80% for patients who received curative resection [2]. Previous studies have reported that some specific factors are associated with the recurrence of HCC, including microvascular invasion (MVI), cytokeratin 19 (CK19) expression, high Ki-67 labeling index and poor histologic differentiation etc. [3–6]. However, these factors can only be identified by the post-operative histopathological examinations. Preoperative identification of patients at high risk for recurrence may improve individualized management and reach better prognosis.

In clinical practice, CT and MRI play an important role in the diagnosis, staging and prognostic evaluation of HCC [7]. However, non-invasive imaging techniques are known to experience difficulties in predicting recurrence of HCC. In addition to morphologic imaging features and dynamic enhancement patterns, tumor stiffness may be used as a prognostic factor, which is regarded as be correlated with the degree of tumor cellularity and fibrosis. Magnetic resonance elastography (MRE) is a useful tool for noninvasive evaluation of tumor stiffness for liver tumor characterization [8]. Previous studies have reported that the MRE-based stiffness could be used as a potential biomarker for predicting HCC recurrence after surgical resection [9]. However, the application of MRE was limited by the need for external mechanical setup and dedicated MRI sequence. Recently, a novel technique named diffusion-weighted imaging (DWI)-based virtual elastography (vMRE) was proposed by Le Bihan et al. [10]. Compared with traditional DWI, the vMRE is proposed on the basis of two b-values (200 and 1500 s/mm^2^). By introducing these higher b-values to the monoexponential model, the vMRE can reveal both Gaussian and non-Gaussian diffusion with theoretically optimized sensitivity. With the enhanced sensitivity to tissue microstructure, previous studies have reported that the so-called shifted apparent diffusion coefficient (sADC) parameter obtained from vMRE has equivalent diagnostic efficacy of liver fibrosis staging as conventional MRE [11]. Moreover, the vMRE can be applied without the need of additional hardware. Since the HCC lesions in patients with recurrence may present with increased cellularity and stiffness, the vMRE might have potential value in the tumor characterization of HCC. To the best of our knowledge, the vMRE has seldom been applied for liver tumors.

Therefore, the purpose of this study is to explore the capability of the parameters obtained from vMRE in the preoperative evaluation of tumor recurrence and underlying relevant histopathological characteristics in HCC patients.

## Materials and methods

This study is a retrospective analysis of patients from a prospective cohort. Our institutional review board approved the analysis of the MRI data and written informed consent was obtained from all patients.

### Patients

**Fig. 1:**
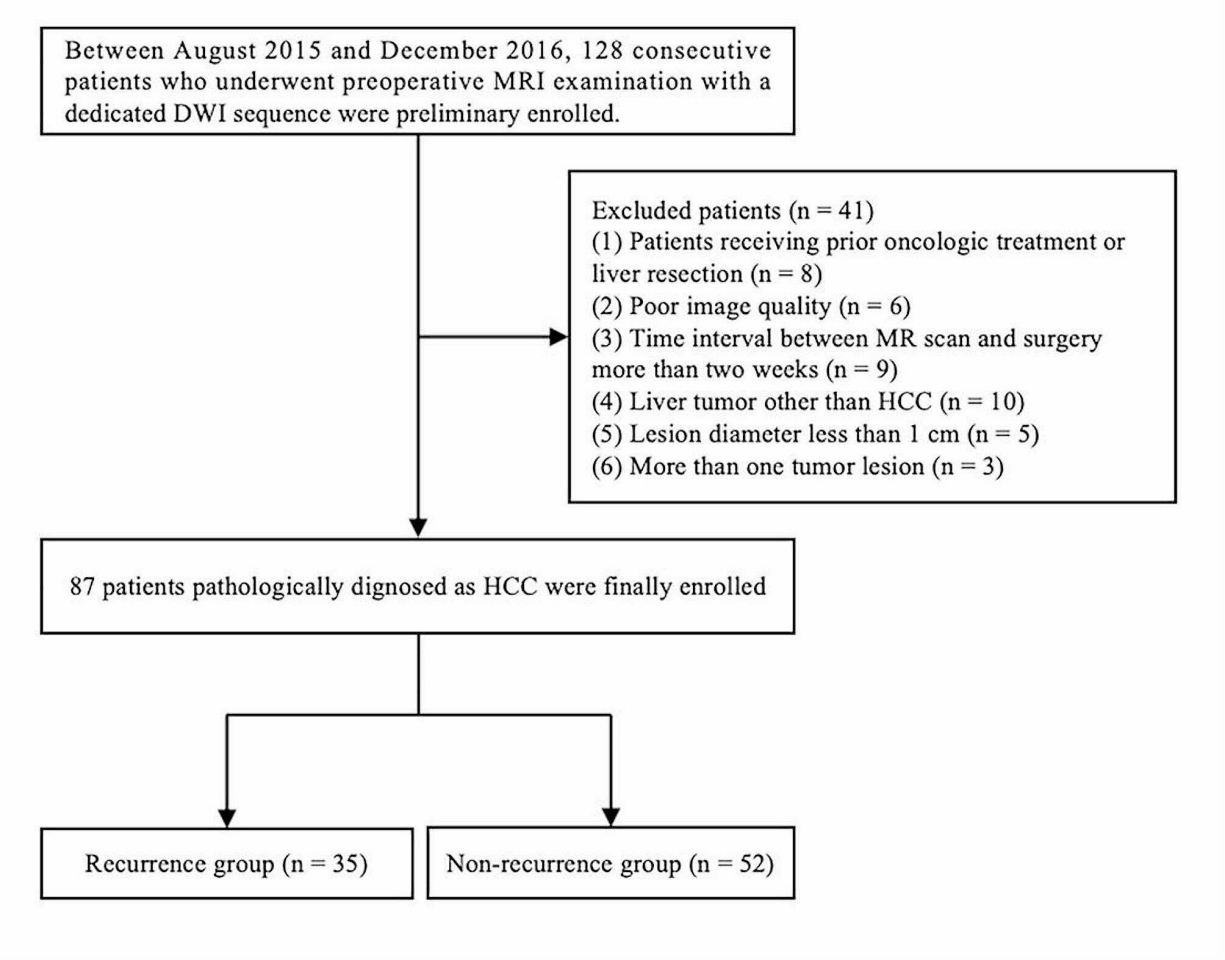
Flow diagram shows inclusion and exclusion criteria in this study MRI: magnetic resonance imaging; HCC: hepatocellular carcinoma

Between August 2015 and December 2016, a total of 128 consecutive patients suspected of hepatic lesions were included. The inclusion criteria were as follows: [1] preoperative DWI sequence including at least 2 b values (200 and 1500 s/mm^2^) and contrast enhanced MR images were acquired; [2] the interval between MR examination and hepatectomy was within 7 days; [3] patients have no prior treatments with hepatic lesions. The exclusion criteria were as follows: (1) histopathological results disprove the diagnosis of HCC; (2) insufficient image quality caused by susceptibility artifacts or respiratory motion; (3) the diameter of lesions was less than 1 cm; (4) patients with more than one HCC lesion. Finally, 87 patients with solitary HCC lesions were enrolled in this study. The flowchart of patient’s selection process is displayed in Fig. 1.

### Imaging acquisition

All MR images were obtained by using a 1.5T MR system (MAGNETOM Aera; Siemens Healthineers, Erlangen, Germany). Diffusion-weighted MR imaging was performed with a single-shot spin-echo echo-planar (ss-EPI) sequence. The detailed parameters were as follows: repetition time (TR) /echo time: 8000 ms/63 ms, field of view, 380 × 308 mm^2^; matrix, 128 × 128; section thickness, 5 mm; b-values: 0, 200, 500 and 1500 s/mm^2^ with a number of averages of 1, 1, 2, 3; diffusion model: 3-scan trace. The total scan time for the DWI protocol was about 2 min and 30s. Other clinical routine MR sequences including a T2-weighted sequence with fat suppression, 3D T1-weighted (in-phase and out-of-phase) volumetric interpolated breath-hold examination (VIBE), and a dynamic 3D T1-weighted VIBE examination was performed before and after the injection of contrast media. The arterial phase, portal venous phase, and equilibrium phase images were obtained at 20–30 s, 70–80 s, and 180 s after injection of 0.1 mmol/kg gadopentate dimeglumine (Magnevist; Bayer Schering Pharma AG, Berlin, Germany) at a rate of 2 mL/sec, respectively. The detailed MR scan parameters are displayed in Table 1.

**Table 1:** The parameters of MRI scan protocol

Parameter	DWI	In/out of phase T1-weighted imaging	T2-weighted imaging	T1-weighted VIBE sequence
Repetition time (ms)	8000	6.87	2400	4.36
Echo time (ms)	63	2.38/4.76	94	2
Field of view (mm^2^)	380 × 308	380 × 278	380 × 308	380 × 297
Scan matrix	128 × 128	320 × 240	320 × 224	320 × 240
Slice thickness (mm)	5	4	5.5	3
Gap (mm)	1	0	1.1	0

DWI: diffusion-weighted imaging; VIBE: volumetric interpolated breath-hold examination

### Imaging analysis

Before ADC and sADC were calculated using an in-house developed program based on MATLAB (Mathworks, Natick, Mass), motion registration was performed for the original DWI images with 4 b-values by using a research application (MR Multiparametric Analysis; Siemens Healthineers). The ADC was calculated based on the following formula:

$$\:\text{A}\text{D}\text{C}\:({\text{m}\text{m}}^{2}/\text{s})\:=\:\text{ln}({S}_{0}/{S}_{500})/500$$

Where S_500_ and S_0_ are the signal intensity when b-values of 500 and 0 s/mm^2^ are applied, respectively.

The shifted apparent diffusion coefficient (sADC) was calculated by using the following formula with two key b values (200 and 1500 s/mm^2^) which was optimized to reflect Gaussian and non-Gaussian diffusion [10].

$$\:\text{s}\text{A}\text{D}\text{C}\:({\text{m}\text{m}}^{2}/\text{s})=\:\text{ln}({S}_{200}/{S}_{1500})/1300$$

Where S_200_ and S_1500_ are the signal intensity when b-values of 200 and 1500 s/mm^2^ are applied, respectively. After sADC was calculated, the diffusion-based shear modulus (*μ*_*diff*_) was then obtained based on the following formula proposed by previous research [10]:

$$\:{\mu\:}_{diff}\:\left(\text{k}\text{P}\text{a}\right)={\alpha\:}\text{s}\text{A}\text{D}\text{C}+\:{\beta\:}$$

where α and β are two constants with values of -12,740 and 14.0, respectively.

All images were analyzed by two independent observers who are blinded to histopathologic and follow-up results. The region of interest (ROI) was outlined in ITK-SNAP for the whole tumor on the DW images of b200, with contrast-enhanced MR images as reference. Furthermore, we also evaluate the background normal hepatic parenchyma by using a total of 3 round ROI of the same extent (100 mm^3^) on the EPI-DWI images of b200, avoiding large vessels and bile ducts, lesions, or artifacts. Then the ROI was simultaneously copied to ADC, sADC and μ maps to generate mean values of ADC, sADC and μ for final analysis. Mean values of the quantitative parameters of the background liver parenchyma were calculated by averaging the measurements of the 3 ROIs. Thirty cases were randomly selected to test the reproducibility of the quantitative parameters.

### MR morphologic features

Two independent radiologists who were blinded to the histopathological results reviewed all MR images in the picture archiving and communication system (PACS). In addition to tumor size, the following morphologic features based on the LI-RADS ver.2018 diagnostic algorithm were evaluated: (1) tumor diameter, (2) tumor margin (classified as smooth margin or non-smooth margin), (3) targetoid appearance on DWI or contrast-enhanced images (including rim arterial phase hyperenhancement, peripheral washout and delayed central enhancement), (4) corona enhancement (defined as the hyperperfusion of liver tissue surrounding the tumor border in late arterial phase or early portal venous phase), (5) intratumoral hemorrhage, and (6) intratumoral fat deposition. The representative images of the MR features are displayed in Supplementary Table S1. When there was a disagreement between the two observers, a consensus would be reached through discussion.

### Histopathological examination and follow-up

Pathological characteristics of surgical resection specimens such as Edmondson-Steiner grade, presence of microvascular invasion (MVI) of tumor, satellite lesions et al. were evaluated and confirmed by a team of experienced pathologists (each with more than 10 years of experience). The expression status of CK19, CD34, CD7 and Gglypican-3 (GPC3) were determined as positive or negative by immunohistochemical staining. In particular, 5% was used as a cutoff value for identifying positive CK19 by using previous research as references [12]. Furthermore, low expression of Ki-67 was defined as ≤ 30% tumor cells were positive, while high expression of Ki-67 was defined as > 30% tumor cells were positive. MVI was defined as tumor emboli in a vascular space lined by endothelium cells on microscopy [13]. The histological grade of HCC was determined according to the Edmondson-Steiner classification and Edmondson-Steiner grade I and II were classified into the low-grade group, grade III and IV were classified into the high-grade group. After surgical resection, patients were followed up with ultrasonography and/or contrasted computed tomography or MRI every 3 months in the first year and every 3–6 months after first year to assess tumor progression. The recurrence-free survival (RFS) was recorded, which was defined as the length of time from the date of hepatectomy to the date of any type of initial tumor recurrence until December 31, 2022.

### Statistical analysis

Continuous variables were analyzed using the independent t-test or Mann-Whitney U test depending on normality test. Categorical variables were analyzed by the chi-square test or Fisher’s exact test. Spearman correlation coefficients were calculated to assess the correlation between quantitative parameters and histologic fibrosis scores. The interobserver agreement was evaluated using intraclass correlation coefficient (ICC) for continuous parameters (≤ 0.2, poor; 0.2–0.4, fair; 0.4–0.6, moderate; 0.6–0.8, good; 0.8–1.0, excellent). The best cutoff value for vMRE-derived parameter was determined based on receiver operating characteristic (ROC) curves. Univariate and multivariate regression analysis was performed to identify independent predictors of recurrence using a Cox proportional hazards model. Univariate analysis was firstly performed and then those parameters showed statistical significance in univariate analysis were used for further stepwise multivariate Cox regression analysis. The Kaplan-Meier method was used to evaluate the recurrence rate and comparison between groups was accessed by using the log-rank test. Finally, the ROC curve and area under the ROC curve (AUC) were used to assess the diagnostic performance of significant variables for predicting recurrence after hepatectomy. The SPSS software (version 26.0) and R software (version 3.6.1) were used for all statistical analysis. A two-sided *P* value less than 0.05 was considered statistically significant.

## Results

### Patient characteristics and follow-up findings

Baseline demographic and clinical characteristics of patients are listed in Table 2. The final study included 87 patients (70 men and 17 women; mean age, 53.7 ± 9.7 years) with solitary HCC. Among all the study population, 35 patients (40.2%) were found to have recurrence after hepatectomy during the whole follow-up period (range 2–60 months; median, 27 months). The median time interval between hepatectomy and HCC recurrence was 18 months (range 2–59 months). The most predominant cause of the underlying liver disease was the chronic hepatitis B viral infection (96.6% of patients). Among pathological features, the HCC lesions in patients with recurrence were more likely to demonstrated MVI (*p* < 0.001) and higher histological tumor grade (*p* = 0.012) than those without. The remaining baseline characteristics of HCC patients with or without recurrence showed no statistical difference.

**Table 2:** Clinical characteristics of 87 HCC patients

Variable	Total (n = 87)	Recurrence (*n* = 35)	Non-recurrence (n = 52)	*P* Value
Gender				0.929
Male	70 (80.5)	28 (80.0)	42 (80.8)	
Female	17 (19.5)	7 (20.0)	10 (19.2)	
Age (years)*	53.7 ± 9.7	54.2 ± 9.4	53.0 ± 10.2	0.565
Total bilirubin > 20.4 µmol/L	13 (14.9)	7 (20.0)	6 (11.5)	0.278
Direct bilirubin > 6.8 µmol/L	28 (32.2)	14 (40.0)	14 (26.9)	0.261
Alanine aminotransferase level > 50 U/L	18 (18.0)	7 (20.0)	11 (21.2)	0.896
Aspartate aminotransaminase level > 40 U/L	27 (31.0)	11 (31.4)	16 (30.8)	0.948
γ-glutamyltransferase > 60 U/L	45 (51.7)	18 (51.4)	27 (51.9)	0.964
AFP > 20 ng/ml	50 (57.4)	17 (48.5)	32 (61.5)	0.232
Carcinoembryonic antigen > 5 ng/ml^**††**^	11 (12.6)	3 (8.6)	8 (15.4)	0.543
Cancer antigen 19 − 9 > 34 U/ml	26 (29.9)	12 (34.3)	14 (26.9)	0.462
Aetiology of liver disease^**†**^				> 0.99
Hepatitis B Virus	84 (96.6)	34 (97.1)	50 (96.2)	
Hepatitis C Virus	1 (1.1)	1 (2.9)	0 (0)	
None or other	2 (2.3)	0 (0)	2 (3.8)	
BCLC stage				0.311
BCLC 0	11 (12.6)	6 (17.1)	4 (1.92)	
BCLC A	76 (87.4)	29 (82.9)	48 (92.3)	
Tumor size^**††**^	4.0 (2.6, 6.0)	4.5 (2.6, 7.3)	4.0 (2.6, 4.9)	0.247
MVI				< 0.001
present	13 (14.9)	12 (34.3)	1 (1.9)	
absent	74 (85.1)	23 (65.7)	51 (98.1)	
Edmondson-Steiner grade				0.012
G1-G2	52 (59.8)	14 (40.0)	35 (67.3)	
G3-G4	35 (40.2)	21 (60.0)	17 (36.7)	
Fibrosis stage				0.341
F0-F2	30 (34.5)	10 (28.6)	20 (38.5)	
F3-F4	57 (65.5)	25 (71.4)	32 (61.5)	

Unless otherwise specified, data are numbers of patients with percentages in parentheses

* Data are means ± standard deviation and compared by using the independent t-test

^**†**^ Data are compared by using the Fisher exact test

^**††**^ Data are means with the interquartile range in parentheses and compared by using Mann–Whitney U test

AFP: alpha-fetoprotein; BCLC: barcelona clinic liver cancer; MVI: microvascular invasion

### Quantitative MR characteristics measurements

**Fig. 2:**
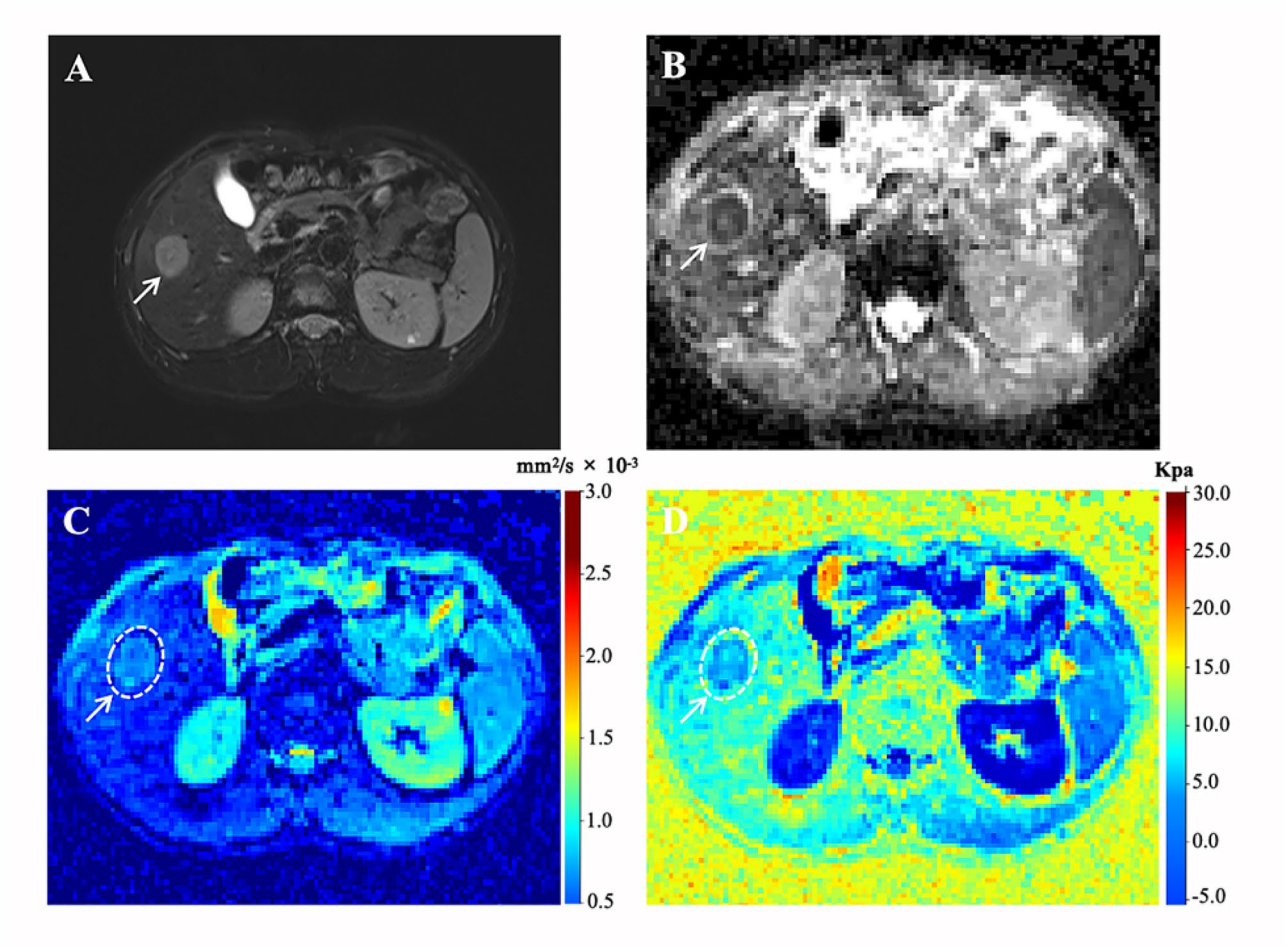
Representative MR images of a 47-year-old man with pathologically verified HCC. Tumor recurrence occurred 13 months after surgery. (**A**) Axial T2-weighted image showing a hyperintensity mass (arrow) on segment VI of the liver. (**B**-**D**) ADC, sADC, and *μ*_*diff*_ maps showing that the mean ADC, sADC, and μ_diff_ values of the tumor (arrow) were 1.44 × 10^− 3^ mm^2^/s, 0.70 × 10^− 3^ mm^2^/s, and 5.10 kPa, respectively. ADC, apparent diffusion coefficient; sADC, shifted apparent diffusion coefficient; *μ*_*diff*_, DWI-based virtual shear modulus

**Fig. 3:**
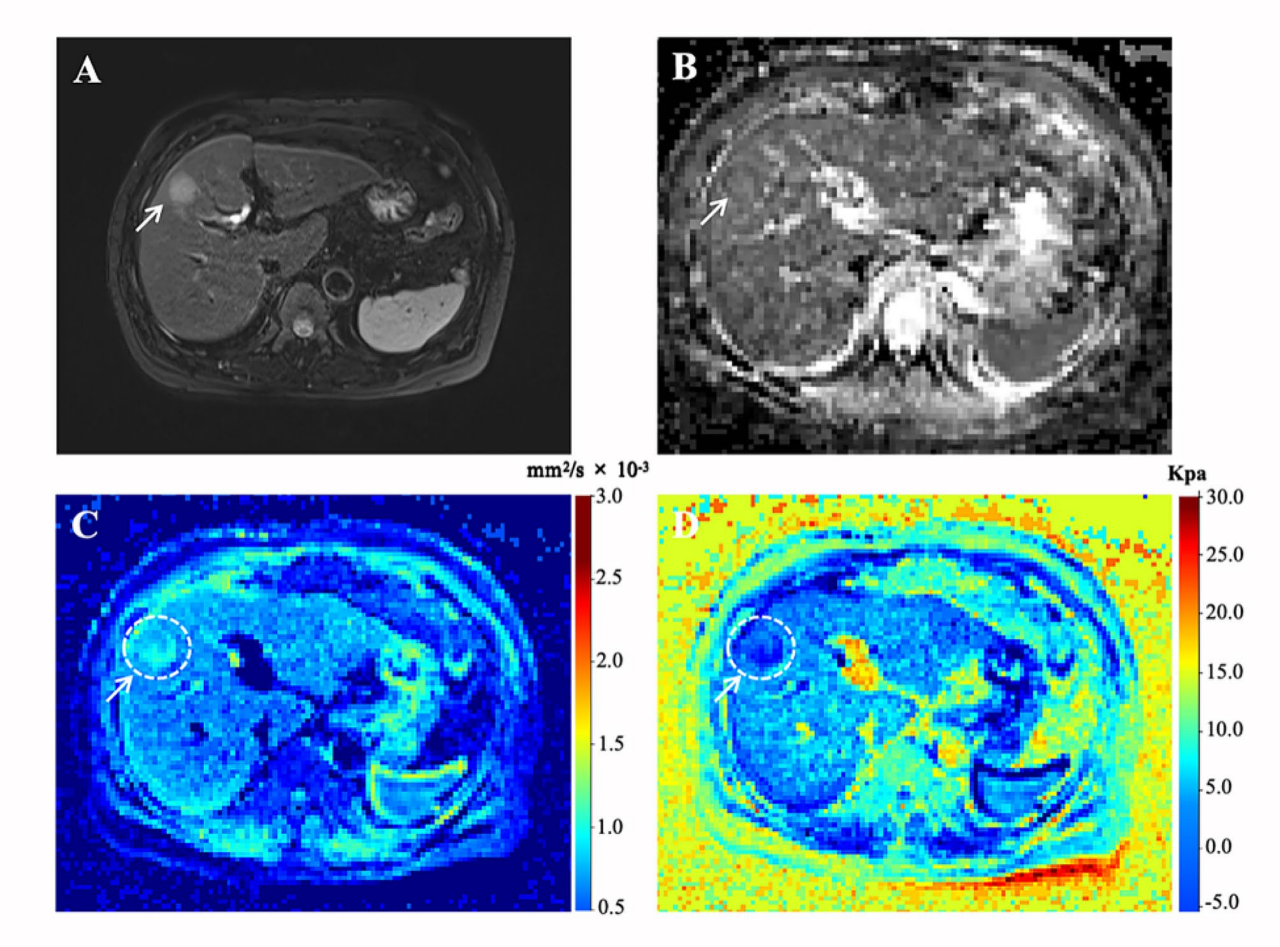
Representative MR images of a 60-year-old woman with pathologically verified HCC without recurrence during a 5-year follow-up. (**A**) Axial T2-weighted image shows a hyperintensity mass (arrow) on segment VIII of liver. (**B**-**D**) ADC, sADC, and μ_diff_ maps showing that the mean ADC, sADC, and μ_diff_ values of the tumor (arrow) were 1.35 × 10^− 3^ mm^2^/s, 0.94 × 10^− 3^ mm^2^/s, and 1.99 kPa, respectively. ADC, apparent diffusion coefficient; sADC, shifted apparent diffusion coefficient; *μ*_*diff*_, DWI-based virtual shear modulus

Among all the quantitative parameters, the HCC lesions in patients with recurrence demonstrated significantly higher *μ*_*diff*_ values compared with those in patients without recurrence (4.27 ± 1.96 vs. 2.60 ± 1.89, *p* < 0.001). However, the ADC values showed no statistical differences between these two groups (1.40, interquartile range (IQR):1.31–1.57 vs. 1.51, IQR:1.30, 1.60, *p* = 0.257) (Figs. 2 and 3). In terms of background liver parenchyma, the *μ*_*diff*_ value of the background liver (*μ*_*diff−liver*_) increased with increasing liver fibrosis stage (rho = 0.563, *p* < 0.001). However, the ADC value was not correlated with liver fibrosis stage (rho = -0.174, *p* = 0.107). The AUC of *μ*_*diff−liver*_ value in diagnosing liver fibrosis stage 3 or greater is 0.778. In the thirty randomly selected cases, the agreements of quantitative parameters between two observers were excellent for ADC, *μ*_*diff*_, ADC_liver_ and *μ*_*diff−liver*_, with ICC of 0.929 (95% confidence interval (CI): 0.860–0.965), 0.935 (0.870–0.969), 0.829 (0.687–0.911) and 0.867 (0.741–0.935), respectively.

### Univariate and multivariate Cox analyses for predictors of RFS

**Fig. 4:**
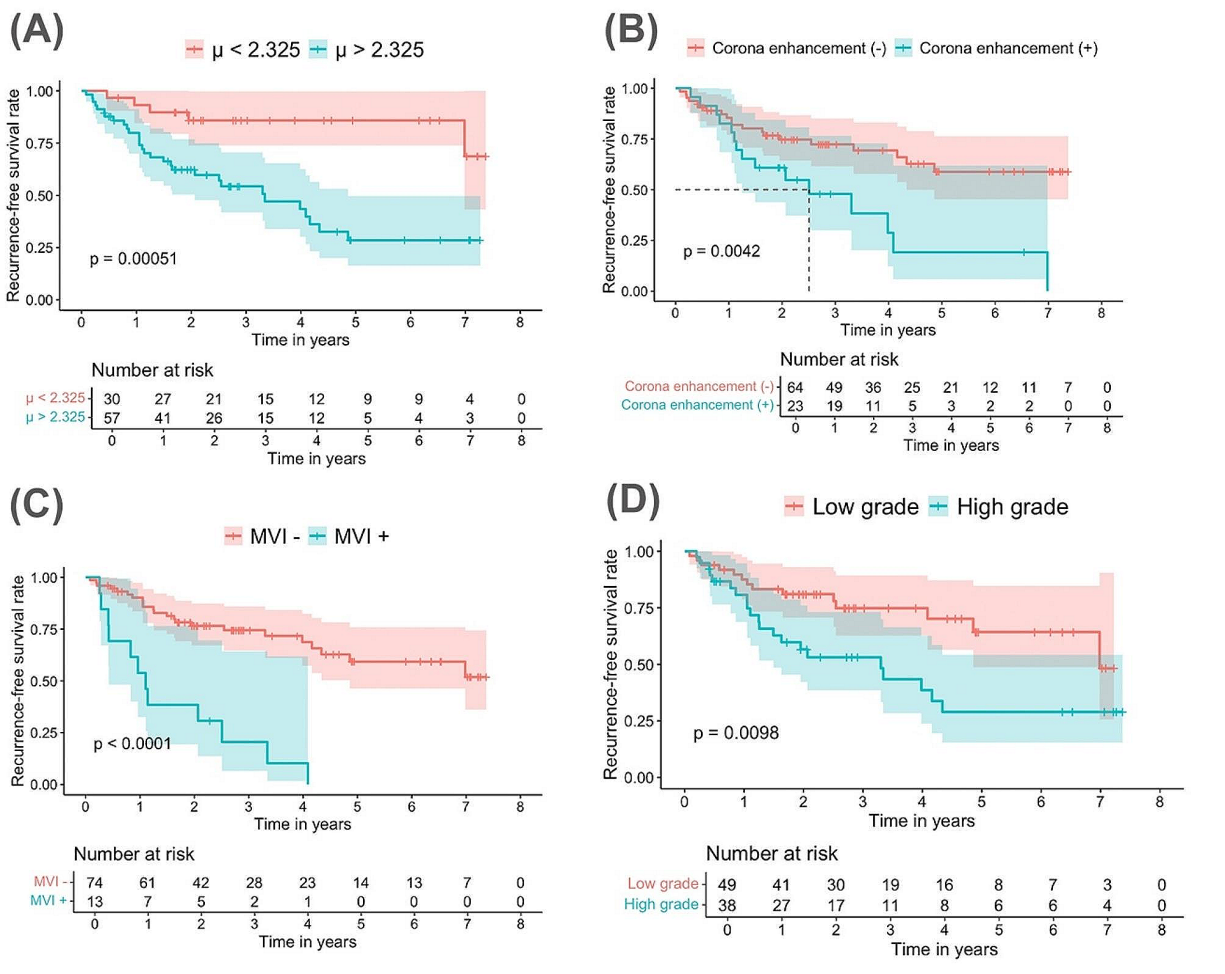
Recurrence-free survival (RFS) outcome. (**a**) The Kaplan–Meier analysis shows that the patients with *μ*_*diff*_ values > 2.325 kPa have lower RFS rates than patients with *μ*_*diff*_ values ≤ 2.325 kPa; (**b**) Patients with corona enhancement HCC lesions have lower RFS rates than patients without corona enhancement HCC lesions. (**c**) Patients with MVI-positive HCC lesions have lower RFS rates than patients without MVI. (**c**) Patients with high histologic tumor grade have lower RFS rates than patients with low histologic tumor grade RFS: recurrence-free survival; ADC: apparent diffusion coefficient; *μ*_*diff*_: DWI-based virtual shear modulus; HCC: hepatocellular carcinoma; MVI: microvascular invasion

**Fig. 5:**
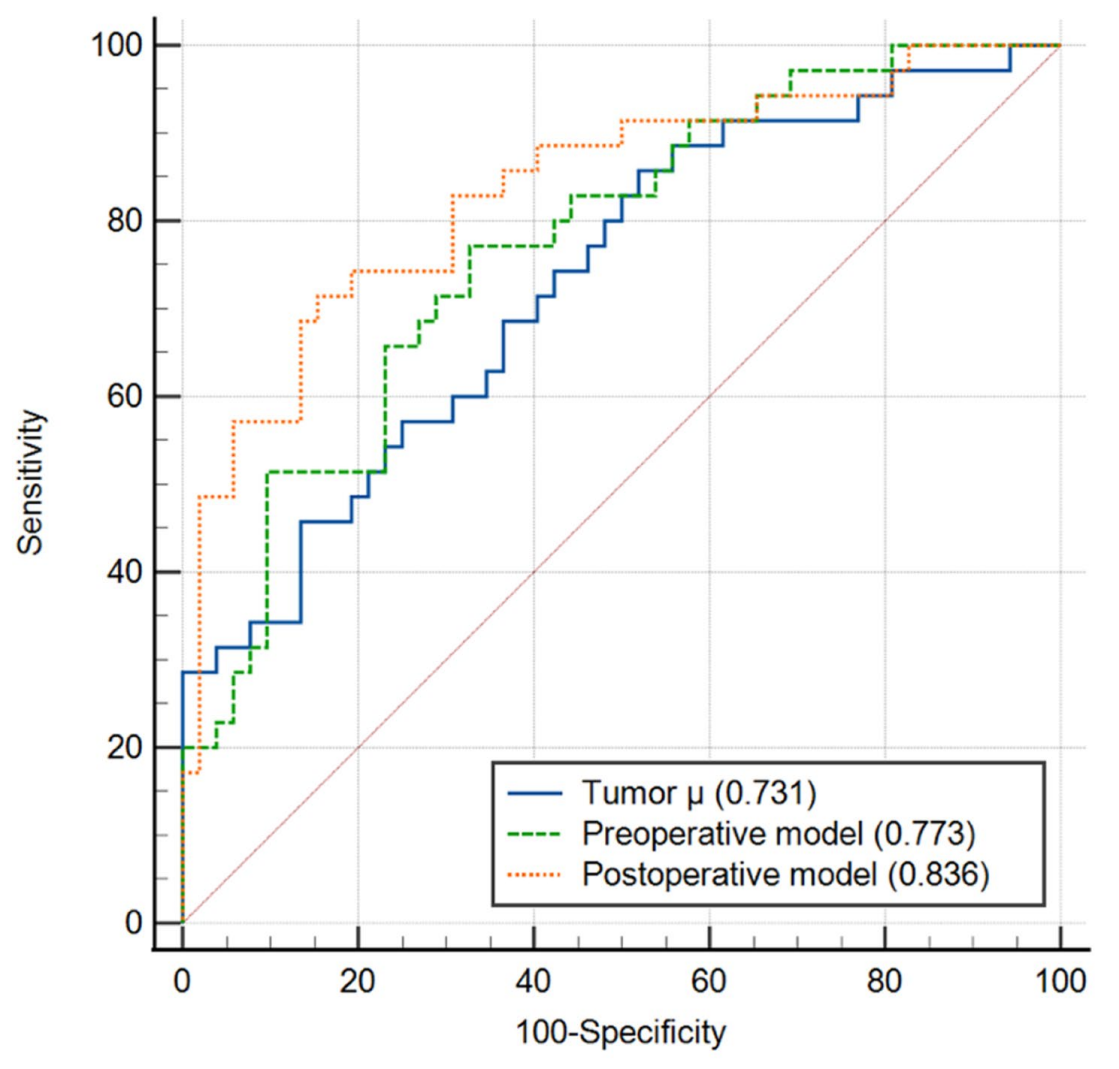
Graph indicating the ROC curves for *μ*_*diff*_, preoperative model and postoperative model for predicting HCC recurrence after hepatectomy. The AUCs for the corresponding ROC curves were 0.731 (*μ*_*diff*_), 0.773 (preoperative model), and 0.836 (postoperative model). AUC: Area under the curve; HCC: hepatocellular carcinoma; ROC: receiver operating characteristic; *μ*_*diff*_: DWI-based virtual shear modulus

In the univariate analysis, the *μ*_*diff*_*values*, corona enhancement, target appearance, tumor grade, MVI and high KI-67 labeling index were significantly associated with RFS (all *p* < 0.05). All the parameters were employed for building the postoperative model including clinic-pathologic and radiologic features. The pathological parameters including tumor grade, MVI and KI-67 labeling index can be obtained only after the operation, therefore they were excluded in the multivariable analysis for establishing the preoperative model. In the subsequent multivariable analysis of Cox proportional hazards regression, independent parameters were identified for both models (Table 3). Since the *μ*_*diff*_ values were identified as the independent risk factors of HCC recurrence in both preoperative and postoperative models, the patients were stratified into a high *μ*_*diff*_ values (> 2.325 kPa) group and a low *μ*_*diff*_ values (≤ 2.325 kPa) group. The Kaplan–Meier method and log-rank test revealed that the 5-year RFS after hepatectomy were significantly shorter in the higher *μ*_*diff*_ values group compared to the lower *μ*_*diff*_ values group. The 1-year, 3-years, and 5-years cumulative recurrence rates of patients with high *μ*_*diff*_ values were 20.1%, 45.7%, 71.5%, respectively and 6.8%, 14.1%, 14.1%, respectively, in the group of patients with low *μ*_*diff*_ values (Fig. 4, *p* < 0.001). The ROC curve of the risk factors selected by Cox regression analysis are displayed in Fig. 5. The AUC of *μ*_*diff*_ values, preoperative model and postoperative model were 0.731, 0.773 and 0.836, respectively. The postoperative model demonstrated the best diagnostic efficiency which is significantly higher than using *μ*_*diff*_ alone (*p* = 0.016). ROC analyses showed that the performance of *μ*_*diff*_ alone in predicting recurrence in HCC was similar to that of the preoperative model (*p* = 0.334) and the pre- and postoperative models showed comparable performance (*p* = 0.137). More details about the diagnostic performance are summarized in Table 4.

**Table 3:** Univariate and multivariate Cox proportional hazards regression analyses of risk factors for recurrence of HCC

Risk Factors	Univariate Analysis			Multivariate Analysis			
				preoperative model		postoperative model	
	*HR (95% CI)*	*p value*		*HR (95% CI)*	*p value*	*HR (95% CI)*	*p value*
Age (years)	1.00 (0.96–1.03)	0.874					
Gender of male	0.51 (0.24–1.12)	0.095					
AFP > 20 ng/ml	0.69 (0.35–1.33)	0.266					
CEA > 5 ng/ml	0.77 (0.24–2.52)	0.667					
CA19-9 > 34 U/ml	1.54 (0.76–3.12)	0.227					
Total bilirubin > 20.4 μmol/L	1.56 (0.60–4.07)	0.359					
Alanine aminotransferase level > 50 U/L	0.87 (0.36–2.10)	0.758					
Aspartate aminotransaminase level > 40 U/L	1.17 (0.58–2.34)	0.674					
Tumor diameter	0.95 (0.85–1.07)	0.428					
Intratumoral hemorrhage	0.51 (0.15–1.66)	0.261					
Intratumoral fat	0.57 (0.17–1.85)	0.347					
Tumor margin	1.98 (0.82–4.76)	0.129					
Corona enhancement	2.61 (1.32–5.17)	**0.006**		2.25 (1.10–4.59)	**0.026**	1.36 (0.57–3.25)	0.483
Targetoid appearance	2.10 (1.06–4.15)	**0.033**		1.70 (0.82–3.53)	0.156	1.53 (0.68–3.41)	0.302
Liver fibrosis stage	1.16 (0.56–2.42)	0.70					
ADC	0.99 (0.99-1.00)	0.340					
ADC_*liver*_	0.99 (0.99-1.00)	0.957					
*μ*_*diff*_	1.39 (1.18–1.64)	**< 0.001**		1.30 (1.09–1.54)	**0.003**	1.23 (1.01–1.50)	**0.037**
*μ*_*diff−liver*_	0.98 (0.80–1.21)	0.874					
CK19	1.80 (0.92–3.51)	0.084					
KI-67	2.59 (1.31–5.13)	**0.006**		NA	NA	1.15 (0.52–2.57)	0.730
MVI	5.65 (2.71–11.78)	**< 0.001**		NA	NA	2.86 (1.10–7.46)	**0.019**
Hitological tumor grade	2.38 (1.21–4.69)	**0.012**		NA	NA	2.07 (1.01–4.23)	**0.047**

AFP: alpha-fetoprotein; ADC: apparent diffusion coefficient; *μ*_*diff*_: DWI-based virtual shear modulus; MVI: microvascular invasion; NA: not applicable

Data in parentheses are 95% CI. Each variable with *P* < 0.05 at univariate analysis was entered into the multivariate analysis

**Table 4:** Diagnostic performance for predicting recurrence of HCC

Parameters	Variables	AUC (95% CI)	Sensitivity (%)	Specificity (%)	Accuracy (%)	PPV	NPV
*μ*_*diff*_	/	0.731 (0.626–0.821)	85.71	48.08	63.22	52.63	83.33
Preoperative model	*μ*_*diff*_, corona enhancement	0.773 (0.670–0.856)	77.01	67.31	71.27	61.36	81.40
Postoperative model	*μ*_*diff*_, MVI and histologic tumor grade	0.836 (0.741–0.907)	71.43	84.62	79.31	75.76	81.48

Data are presented as percentages. Data in parentheses are the number of subjects used to calculate the percentage

*μ*_*diff*_: DWI-based virtual shear modulus. AUC: areas under the ROC curve; 95% CI: 95% confidence interval; PPV: positive predictive value; NPV: negative predictive value. MVI: microvascular invasion

### Histopathological characteristics

**Fig. 6:**
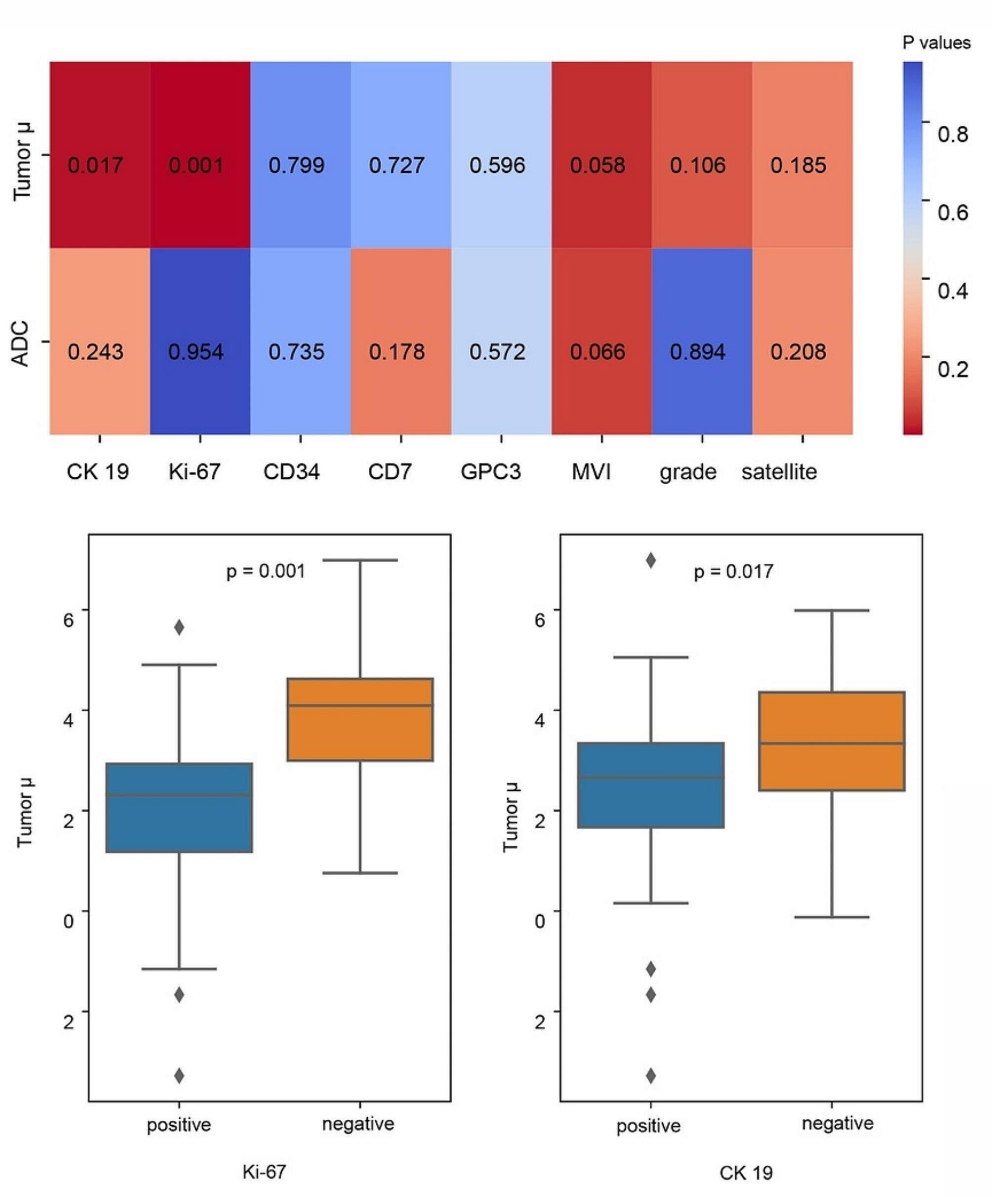
The correlation between quantitative parameters and histopathological characteristics of HCC. (**a**) The heat map shows the *P* values of both ADC and *μ*_*diff*_ values in HCC lesions with or without different histopathological characteristics. (**b**) The Box-and-whisker plots show distributions of *μ*_*diff*_ values in HCC lesions with or without expression of CK19 and high Ki-67 labeling index of HCC. ADC: apparent diffusion coefficient; *μ*_*diff*_: DWI-based virtual shear modulus; HCC: hepatocellular carcinoma

The correlations between the *μ*_*diff*_ values and histopathological characteristics of HCC lesions are showed in Fig. 6. The mean values of *μ*_*diff*_ of CK19-positive HCCs were significantly higher than the CK19-negative HCCs (3.95 ± 2.37 vs. 3.15 ± 1.77, *p* = 0.017). In addition, the HCC lesions with high expression of Ki-67 also showed significantly higher *μ*_*diff*_ values (4.22 ± 1.63 vs. 2.72 ± 2.12, *p* = 0.001). There were no statistical differences of the *μ*_*diff*_ values in the HCC lesions classified by the remaining histopathological characteristics.

## Discussion

In this study, our results demonstrated that the *μ*_*diff*_ values and corona enhancement were independent risk factors for predicting RFS of HCC patients. More pertinently, we found that the HCC patients with higher *μ*_*diff*_ values and corona enhancement tended to have a higher incidence of HCC recurrence. Furthermore, the higher *μ*_*diff*_ values were associated with expression of CK19 and high Ki-67 labeling index of HCC.

Previously published studies emphasized the value of tumor stiffness evaluated by MRE for prediction of the tumor recurrence of HCC after hepatectomy [9, 14, 15]. Wang et al. [14] reported that tumor stiffness measured by MRE was independently associated with early recurrence of HCC. In addition, Park et al. [15] also revealed that the MRE-based tumor stiffness was a significant predictive factor for RFS of HCC patients after hepatectomy. In theory, tumor invasion and metastasis in HCC are facilitated by higher tumor stiffness, which is driven by increased extracellular matrix (ECM) rigidity due to excessive collagen deposition and crosslinking of extracellular matrix proteins [16, 17]. The ECM plays an important role in promoting tumor cell proliferation, differentiation and chemotherapeutic resistance, resulting in increased tumor stiffness and poor prognosis [18]. Therefore, preoperative evaluation of tumor stiffness might have potential benefits for improving long-term prognosis of HCC patients. However, clinical application for MRE is limited due to the need for extra equipment, the dedicated MRI sequence and longer acquisition times. In recent years, studies showed the possibility of the DWI-based virtual MRE for assessment of liver fibrosis [10, 11], which was also confirmed in the present study. Ota T et al. [19] demonstrated there were strong correlations between sADC values and the MRE-based stiffness values both in the liver parenchyma and liver tumors [19], which suggests the possibility of using the vMRE-derived *μ*_*diff*_ values for preoperative evaluation of tumor characteristics of HCC. In the present study, we explored the relationship between the vMRE parameters and prognosis of HCC patients and found higher *μ*_*diff*_ values were an independent risk factor for predicting RFS of HCC patients after surgical resection. We speculate this might be attribute to the higher stiffness caused by the significant cell proliferation in more aggressive HCC [20]. The use of vMRE as a complement to MRE is intriguing and may have potential clinical value in treatment optimization for HCC patients. In contrast to MRE, the vMRE can be easily incorporated into routine MRI scan protocols with a simple workflow and short acquisition time.

Another interesting result of this study is that the higher *μ*_*diff*_ values were found to be associated with the expression of CK19 and high Ki-67 labeling index of HCC. The CK19-positive HCC shows abundant fibrous stroma which may result in increased cellularity and stiffness [21]. The Ki-67 labeling index can reflect the level of tumor proliferation. The HCC lesions with high Ki-67 labeling index showed faster cell proliferation than those with low Ki-67 labeling index during tumorigenesis, which resulted in increased nucleus/cytoplasm ratio and decreased extracellular/intracellular space. Both of these two histopathological characteristics can lead to increased *μ*_*diff*_ values, which have the potential to noninvasively reveal the cellularity and stiffness in HCC lesions. The association between *μ*_*diff*_ values and the expression CK19 and high Ki-67 labeling index of HCC lesions might explain why HCC patients with higher *μ*_*diff*_ values have a shorter RFS. The mean ADC values in our study had no correlation with tumor recurrence which is in accordance with studies reported by Chuang et al. [22] and Nakanishi et al. [23]. The ADC values derived from conventional monoexponential diffusion with two relatively low b-values was based on the assumption of the simple Gaussian diffusion behavior, which is inherently defective for heterogeneous tumor tissues. This might be the reason why previous research indicated controversial conclusions in the prediction of HCC recurrence [22–24]. By using higher b-values (b = 200, 1500 s/mm^2^), the vMRE can reflect both Gaussian and non-Gaussian diffusion with increased sensitivity to tissue microstructure, thereby reaching better diagnostic efficiency in the prediction of prognosis of HCC.

The value of morphological features in MRI in prediction of prognosis of HCC patients have been widely reported. In our study, we found that the corona enhancement was an independent risk factor for predicting HCC recurrence in preoperative model. This finding was consistent with research reported by Wei et al. [25] and An et al. [26]. The presence of corona enhancement is caused by disordered venous drainage that develops during tumorigenesis due to obstruction of intratumoral hepatic veins, indicating a propensity to infiltrate drainage vessels of the tumor, leading to intrahepatic metastases [27–29]. The MVI and histologic grade has been widely reported and established as significant risk factors of postoperative recurrence [30, 31], which was also confirmed in the present study. By adding the pathological characteristics, the postoperative model demonstrated best diagnostic efficiency which is significantly higher compared with using *μ*_*diff*_ alone. The pre- and postoperative models showed comparable performance for predicting RFS. This may render the findings more meaningful in clinical practice. The patients in the very early stage who received radiofrequency ablation without pathological information might benefit from the preoperative model by allowing reappraisal of tumor biology. However, the value of vMRE parameter in predicting the prognosis of patients with different therapeutic tools for HCC need further study in the future.

Our study had several limitations. First, the retrospective nature of this study may have introduced a selection bias and the sample size is relatively small. Second, a previous study reported by Hanniman et al. [32] found that the fat-corrected vMRE was not associated with fibrosis stage in Non-alcoholic fatty liver disease. Since the most of the study population were infected by HBV, whether the vMRE can be used for predicting prognosis in HCC patients caused by other etiologies need further investigation. Third, the patients in this study did not take the MRE examination, and the direct correlations between the *μ*_*diff*_ values and MRE-based stiffness values of the tumors should be testified in a larger prospective cohort. Finally, the identified optimal cut-off values of the *μ*_*diff*_ values in our study were not tested in a separate validation cohort due to relatively small sample size. Future studies with larger number of patients in a multicenter setting are warranted.

## Conclusion

In conclusion, the vMRE is a promising tool for prediction of HCC recurrence after hepatectomy. Patients with higher μ_diff_ values may need more regular follow-up to detect tumor recurrence in the early stage, thus achieving a better prognosis.

## Electronic supplementary material

Below is the link to the electronic supplementary material.

Supplementary Material 1

## Data Availability

The datasets used and/or analysed during the current study are available from the corresponding author on reasonable request.

## Abbreviations

AUC: Area under the receiver operating characteristic curve
CK19: Cytokeratin 19
HCC: Hepatocellular carcinoma
HR: Hazard ratio
RFS: Recurrence-free survival
sADC: Shifted apparent diffusion coefficient
vMRE: Diffusion–based virtual MR elastography
