# Supplementary material for: Diffusion–based virtual MR elastography for predicting recurrence of solitary hepatocellular carcinoma after hepatectomy
Source: Cancer Imaging. 2024 Aug 13;24:106. doi: 10.1186/s40644-024-00759-8 (PMC11320769; doi:10.1186/s40644-024-00759-8)
Supplement: Supplementary file 1 — Supplementary Material 1 [file 40644_2024_759_MOESM1_ESM.docx]

**Supplementary Table S1.** Definitions and representative images of MRI features

| MRI feature | Definition | Example | |
| --- | --- | --- | --- |
| Diameter | Largest outer-edge-to-outer-edge dimension of observation. | 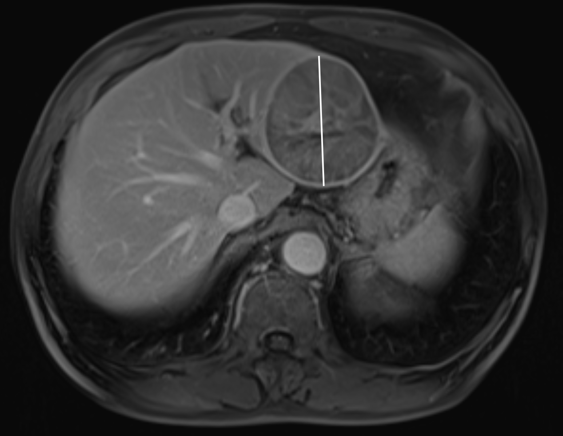 | • Include “capsule” in measurement.  • Pick the phase, sequence, and plane in which margins ar­­e clearest. |
| Tumor margin | Classified as smooth margin or non-smooth margin. | 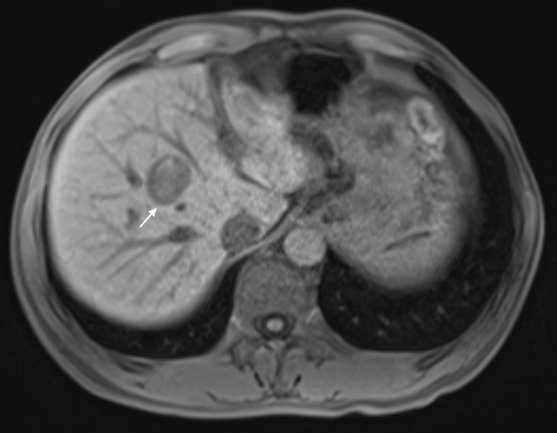 | 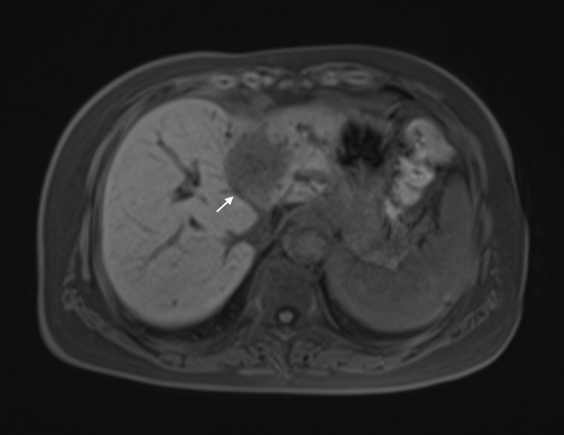 |
|  |  | Smooth margin | Non-smooth margin |
| Rim APHE | A spatially defined subtype of APHE in which arterial phase enhancement is most pronounced in the observation periphery | 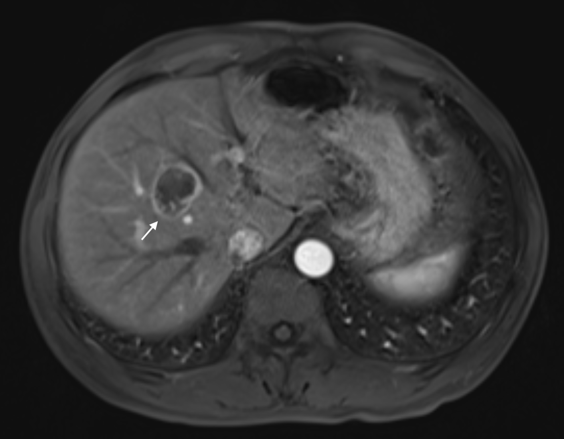 | 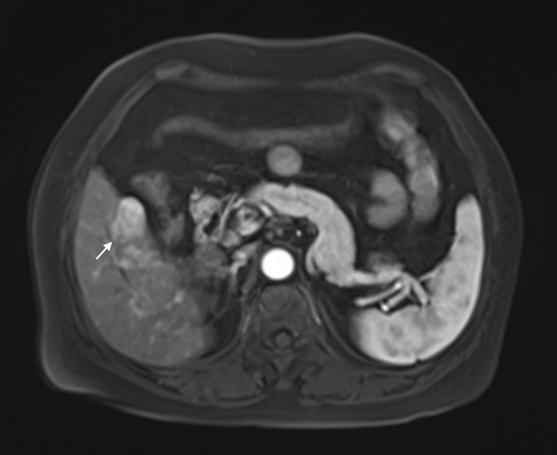 |
|  |  | Present | Absent |
| Peripheral “washout” | Spatially defined subtype of “washout” in which apparent washout is most pronounced in observation periphery | 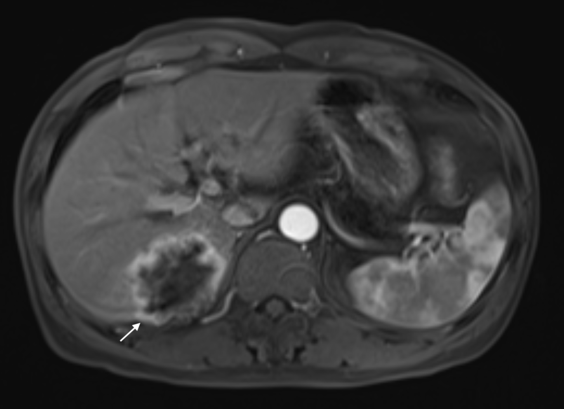 | 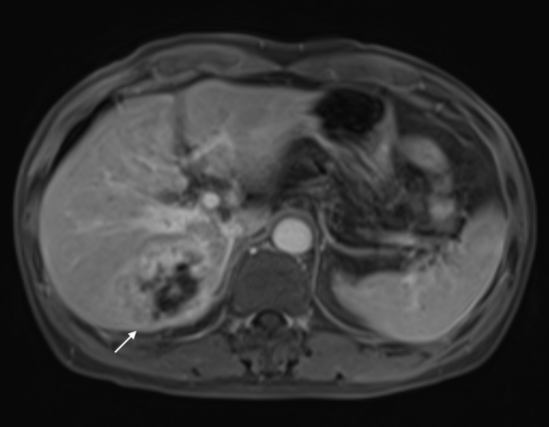 |
|  |  | Arterial phase | Venous phase |
| Delayed central enhancement | Central area of progressive postarterial phase enhancement. | 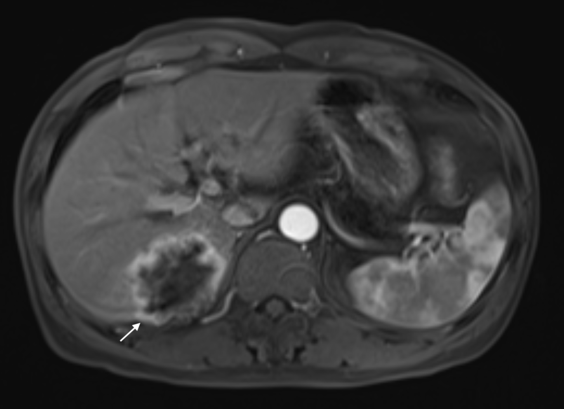 | 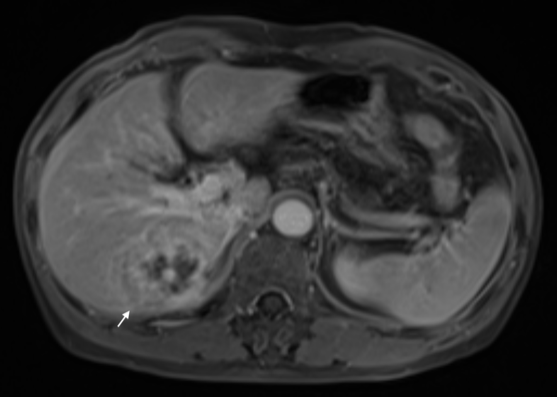 |
|  |  | Arterial phase | Delayed phase |
| Targetoid restriction | Concentric pattern on DWI characterized by restricted diffusion in observation periphery with less restricted diffusion in observation center | 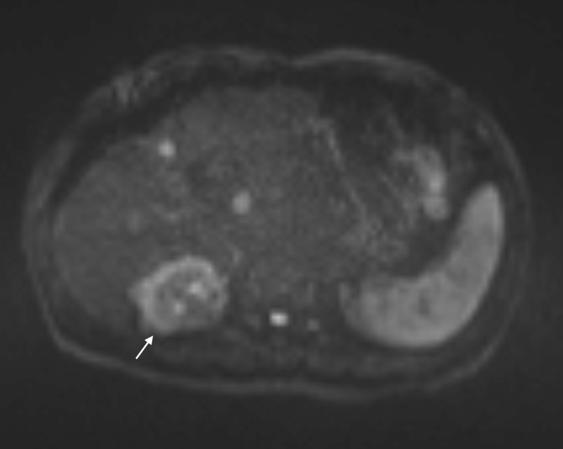 | 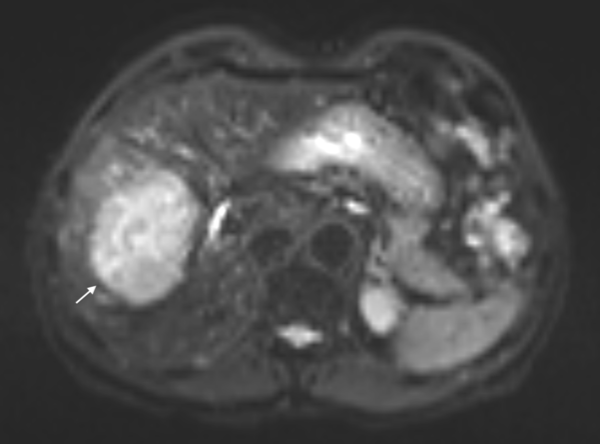 |
|  |  | Present | Absent |
| Corona enhancement | The hyperperfusion of liver tissue surrounding the tumor border in late arterial phase or early portal venous phase. | 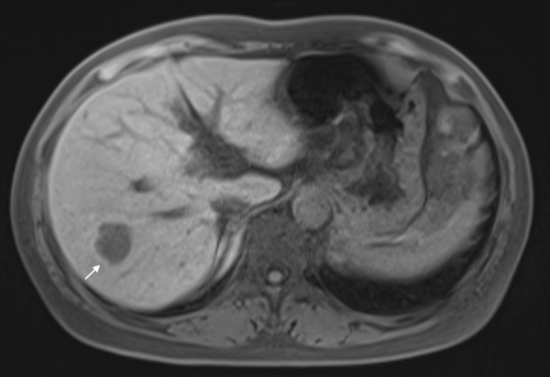 | 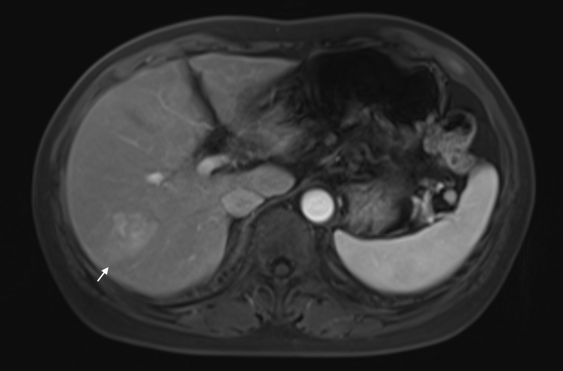 |
| Non-enhancing “capsule” | The intensity in the T1-weighted is unequivocally high relative to adjacent tumor. | 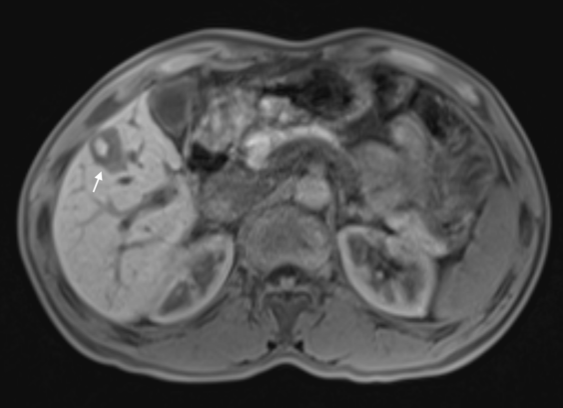 | 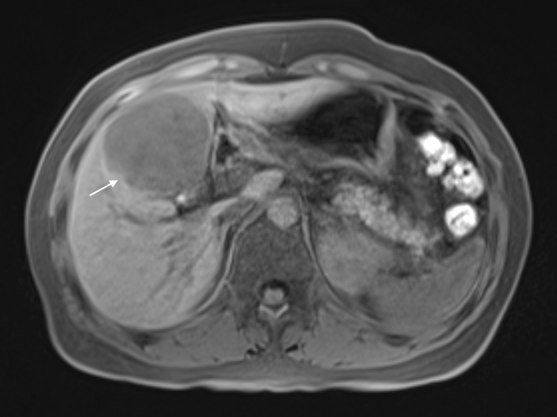 |
| Fat sparing in a solid mass | Tumor area with decreased signal intensity on opposed-phase images compared with in-phase images. | 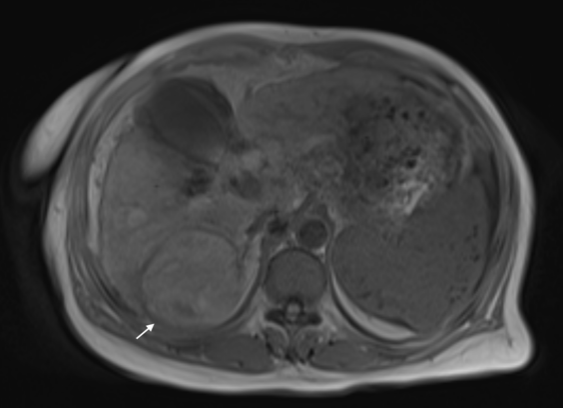 | 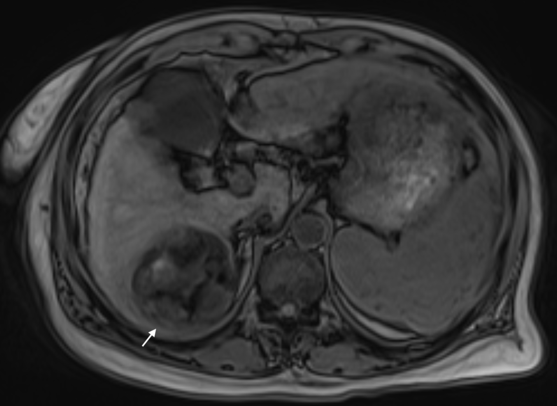 |
|  |  | In-phase | Out-phase |
